# Improving quality of life in the elderly: hearing loss treatment with cochlear implants

**DOI:** 10.1186/s12877-023-04642-2

**Published:** 2024-01-04

**Authors:** D. Cuda, M. Manrique, Á. Ramos, M. Marx, R. Bovo, R. Khnifes, O. Hilly, J. Belmin, G. Stripeikyte, P. L. Graham, C. J. James, P. V. Greenham, I. Mosnier

**Affiliations:** 1 Ospedale Guglielmo da Saliceto, Piacenza, Italy; 2 Clinica Universitaria de Navarra, Pamplona, Spain; 3 Complejo Hospitalario Universitario Insular Materno Infantil, Las Palmas de Gran Canaria, Spain; 4 Hôpital Purpan, CHU, Toulouse, France; 5 Azienda Ospedaliera di Padova, Padova, Italy; 6 Bnai Zion Medical Center, Haifa, Israel; 7 Sackler Faculty of Medicine, Rabin Medical Center, Tel Aviv University, Petah Tikva, Israel; 8 Sorbonne Université Pierre and Hôpital Charles Foix, Paris, France; 9 Cochlear AG, Basel, Switzerland; 10 Macquarie University, Sydney, Australia; 11 Cochlear France SA, Toulouse, France; 12 Greenham Research Consulting Ltd, Ashbury, SN6 8LP UK; 13 Unité Fonctionnelle Implants Auditifs, ORL, GH Pitié-Salpêtrière, AP-HP Sorbonne Université - Technologies et thérapie génique pour la surdité, Institut de l’audition, Institut Pasteur/Inserm, Université Paris Cité, Paris, France

**Keywords:** Cochlear implant, Health utility, Hearing, Hearing aid, Quality of life, Outcomes, Cognition, Healthy aging

## Abstract

**Background:**

Hearing loss impacts health-related quality of life and general well-being and was identified in a Lancet report as one of the largest potentially modifiable factors for the prevention of age-related dementia. There is a lack of robust data on how cochlear implant treatment in the elderly impacts quality of life. The primary objective was to measure the change in health utility following cochlear implantation in individuals aged ≥ 60 years.

**Methods:**

This study uniquely prospectively recruited a large multinational sample of 100 older adults (mean age 71.7 (SD7.6) range 60–91 years) with severe to profound hearing loss. In a repeated-measures design, pre and post implant outcome measures were analysed using mixed-effect models. Health utility was assessed with the Health Utilities Index Mark III (HUI3). Subjects were divided into groups of 60–64, 65–74 and 75 + years.

**Results:**

At 18 months post implant, the mean HUI3 score improved by 0.13 (95%CI: 0.07–0.18 p < 0.001). There was no statistically significant difference in the HUI3 between age groups (F[2,9228] = 0.53, p = 0.59). The De Jong Loneliness scale reduced by an average of 0.61 (95%CI: 0.25–0.97 p < 0.014) and the Lawton Instrumental Activities of Daily Living Scale improved on average (1.25, 95%CI: 0.85–1.65 p < 0.001). Hearing Handicap Inventory for the Elderly Screening reduced by an average of 8.7 (95%CI: 6.7–10.8, p < 0.001) from a significant to mild-moderate hearing handicap. Age was not a statistically significant factor for any of the other measures (p > 0.20). At baseline 90% of participants had no or mild depression and there was no change in mean depression scores after implant. Categories of Auditory perception scale showed that all subjects achieved a level of speech sound discrimination without lip reading post implantation (level 4) and at least 50% could use the telephone with a known speaker.

**Conclusions:**

Better hearing improved individuals’ quality of life, ability to communicate verbally and their ability to function independently. They felt less lonely and less handicapped by their hearing loss. Benefits were independent of age group. Cochlear implants should be considered as a routine treatment option for those over 60 years with bilateral severe to profound hearing loss.

**Trial Registration:**

ClinicalTrials.gov (http://www.clinicaltrials.gov/), 7 March 2017, NCT03072862.

## Introduction

Healthy aging was redefined by the World Health Organization (WHO) in 2015 as more than just the absence of disease but the maintenance of functional ability that enables wellbeing [1]. This means that people should have the capabilities to be and do what they have reason to value and includes a person’s ability to build and maintain relationships and contribute to society.

The recent World Report on Hearing suggests that over 65% of those over 60 years old have some degree of disabling hearing loss and the degree of this hearing loss increases exponentially with age [2]. The impact that even mild to moderate hearing loss, when unaddressed, has on individual’s ability to enjoy life to the full should not be underestimated: As well as listening and communication, hearing loss can affect mental health, social integration and employment [3–5]. It has also been identified as the largest potentially modifiable risk factor for age-related dementia [6]. Its prevalence and its impact on quality of life is why age-related hearing loss was identified as one on the top ten contributors to the increased global burden of disease in those over 50 years old [7]. However, the effects of hearing loss can be reduced with rehabilitation and appropriate amplification with hearing aids or cochlear implants (CI) [2, 6, 8].

Cochlear implants are electronic devices that stimulate the auditory nerve directly via electrodes placed into the cochlea. They are an important part of addressing age related hearing loss for those where hearing aids do not provide sufficient amplification. They are well-established as a safe and effective therapy for severe to profound hearing impairment and provide benefit for patients of all ages, including the elderly [9, 10]. They help to restore hearing function and communication abilities and may even delay cognitive decline [11]. Cochlear implants improve recipients’ quality of life, but despite the benefits that contribute towards the WHO healthy aging aims, less than 10% of adults and less than 1% of older adults who could benefit receive a CI [12, 13]. There are multiple reasons for this, including lack of awareness of the benefits of CI treatment from policy makers and health care professionals and attitudes towards age related deafness in adults [12]. Hearing loss should not be considered an acceptable part of aging. The World Report on Hearing emphasises the urgency of acting to reduce the impact of hearing loss on quality of life, including in the elderly [2].

Studies reporting quality of life improvements in adults with CIs have tended to focus on disease specific measures, rather than on generic measures or health utility [13, 14]. Although disease specific measures maybe more sensitive to quality of life changes, they do not allow for comparisons across different medical treatments. Robust measures of health utility are needed to enable health care professionals, market regulators, and health policy makers, to make informed decisions about the provision of cochlear implants and achieve fairer access to treatment. Standardised health utility measures assess the changes in quality of life as a result of a treatment. They are used in cost effectiveness analysis and are essential to assess the economic impacts of CI and to place the costs into context against other medical treatments [14, 15].

The Health Utility Index Mark 3 (HUI3) is the generic health utility questionnaire that is considered most sensitive to hearing loss and is generally used in CI studies [16]. In a recent meta-analysis, Dixon et al. (2023) identified 18 studies using the HUI3 in adults receiving a unilateral cochlear implant, but only one of these studies reported age as a characteristic [17]. Few other studies have reported HUI3 scores in cohorts of adults in the older age range (60 or older) and these are mainly underpowered based on the known variation in HUI3 scores [14, 17–20]. One exception was Wick et al. (2020), who reported mean multi-attribute HUI3 scores for 70 subjects aged 65 and older. This was a subgroup analysis of a larger study, where the primary outcome measure was speech perception and a clinically meaningful improvement in both speech perception and quality of life was observed [19]. Although post operative speech perception scores in older recipients are lower than for younger recipients, gains in speech perception and quality of life are equivalent across age groups [21–24].

There is also a paucity of data on the impact of implantation in older adults on psychosocial factors that contribute to healthy aging such as loneliness and independence [4, 25]. Social isolation has been strongly linked with dementia and reduced loneliness due to better hearing may reduce cognitive decline [11].

The study protocol is described in full by Marx et al., 2020 [26]. This paper reports on the primary objective of this study, which was to measure the impact of cochlear implantation on health utility in individuals aged ≥ 60 years. To understand whether age impacts benefit, we further divided the cohort into three groups of 60–64, 65–74 and 75 + years. Secondary outcomes relating to psychosocial functions that contribute to quality of life measures are also reported. These included loneliness, depression, and independence. Other measures will be reported in a further paper.

## Method

This was a multi-national observational study, using a repeated-measures, single-subject design in which each subject acts as his/her own control. Subjects were evaluated as part of their routine clinical visits. Each subject was assessed during three visits: Baseline (< 2 months before cochlear implant surgery) and at 12- and 18-months post-surgery. The protocol allowed for appointments to be ± one month from the scheduled visit. The full battery of tests was repeated at each of the three visits. Recruitment was from November 2017 to March 2022.

The implant clinics were chosen for their experience and existing capacity to recruit and treat elderly CI candidates within a reasonable time frame for the study. All subjects who had been assessed as suitable for a CI and had already decided to proceed with a CI manufactured by Cochlear Ltd and met the study criteria were invited to participate.

Subjects were required to be ≥ 60 years old, based on the definition of old by the United Nations and be unilateral CI candidates with bilateral post linguistic onset of moderately-severe to profound deafness, and who met all local criteria for CI treatment. Full criteria for study participation are given in an earlier publication [26].

All enrolled subjects independently gave their written informed consent for participation in the study.

### Measures

Outcomes from routine practice were recorded using clinically well-established scales, widely available in geriatric and audiology practices. Subjects were evaluated in their native languages (Italian, French, Spanish, Arabic and Hebrew). Certificated forwards/backwards translation was carried out by external professional translation providers. A full description of the protocol can be found in Marx and colleagues [26].

Data was collected from the sound processor via its inbuilt datalogging software to evaluate device use.

#### Health utility

The “four-week recall” version of the self-assessed Health Utility Mark 3 (HUI3) (available to purchase from Health Utilities Inc.) questionnaire with 15 questions was completed by the patient [27]. HUI3 multi attribute scores of less than 0.7 are considered to indicate severe disability, between 0.7 and 0.88 moderate disability, and 0.89 or better a mild or no disability. A change of 0.03 or more between time points is clinically significant. Validated non-English language versions of the HUI3 were purchased where required.

#### Depression

The Geriatric Depression Scale-15 (GDS-15) was completed by the patient [28]. This is a 15 question yes/no self-report questionnaire of depressive symptoms in older adults. Scores of 0–4 are considered normal, scores of 5–8 indicate mild depression; 9–11 indicate moderate depression; and 12–15 indicate severe depression. A cut-off of 5 on this screening measure has been shown to indicate depression with a sensitivity of 71.8%, and specificity 78.2% [28].

#### Dependency

The Lawton Instrumental Activities of Daily Living Scale (IADL) is a tool completed by the clinician [29]. It determines the patient’s ability to care for him or herself and was completed using patient hospital files and interview. Scores are based on what an individual can do without human help rather than what they are doing. A lower score indicates a higher level of dependence. Scores range from zero to eight.

#### Loneliness

The six item De Jong Loneliness scale (DJLS) assessed social and emotional isolation [30]. The was either administered in a face-to-face interview, by telephone interview, as self-administered (mail) questionnaire or via electronic data collection. Scores range from 0 not lonely to 6 intensely lonely. Scores of 0 and 1 are considered as “not lonely”; those > 1 as “lonely”.

#### Subjective hearing performance

The Hearing Handicap Inventory in the Elderly Screening test (HHIE-S) was completed by the patient [31]. It consists of 10 questions with yes, sometimes and no answers scored as four, two and zero points respectively. Possible scores range from 0 (no handicap) to 40 (maximum handicap). A change of > 9.3 points at the 95% confidence interval indicates clinical significance [32].

The Categories of Auditory Perception II (CAP-II) is a hearing skill rating scale consisting of nine hierarchical categories [33, 34]. The CAP-II is completed by the clinician as an observation of the individual’s hearing abilities. Ranging from 1 to 9, the auditory skills increase in complexity ranging from perception of environmental sounds to telephone conversation with an unfamiliar speaker. Although mainly used in children, the CAP has also been validated for use in adults [35]. The CAP II adds two additional levels to the CAP and has also been used in adults [34, 36].

### Statistics

A power calculation indicated that a minimum of 68 subjects was required to find a significant difference of 0.1 units for the HUI3 multi-attribute index at the 18 month time point using a paired t-test with a significance level of 5% and power of 90%. Though the 18 month difference was of primary interest, if Bonferroni methods were used to adjust for all pairwise comparisons then a sample size of 88 would be needed to find a difference of 0.1 units significant, as such a planned sample size of 100 guarded against issues with multiple comparisons and subject dropout.

Outcome data were analysed using mixed-effect models (MEM) where interest was in change over time after adjusting for age group (young old defined as 60–64, middle old 65–74 and old old 75 + years). Data lost to follow-up are missing at random. As such all models used all available visits (baseline, 12 months and 18 months post implant) and age group as fixed effects and subject as a random effect to control for the repeated measures. Linear MEMs were used for continuous independent variables (HUI3 multi-attribute score, HHIE-S, GDS-15 (log score) and ordinal MEMs for ordered scales with a limited number of intervals (HUI3 speech and hearing subscales, IADL, DJLS, CAP-II). Model assumption checks included visual inspection of normal quantile plots to assess normality of the errors and random effects. Tukey pairwise comparisons were used to compare all pairs of time points. A 5% significance level was used throughout.

## Results

### Participants

One hundred subjects were originally recruited. However, there were two subjects who did not meet inclusion/exclusion criteria and were excluded from the analysis. Demographics of the 98 subjects are outlined in Table 1. The Mini Mental State Examination screening test for cognitive function showed that 75% of subjects had no cognitive impairment and 22% mild cognitive impairment, 1% had moderate cognitive impairment and 2% were missing. There was no follow-up data for seven subjects. Reasons given for withdrawal were lost to follow up (n = 3), protocol deviation (n = 2), consent withdrawn (n = 1), and investigator decision (n = 1). Thirteen data points were missing at 12 months post implant and 16 at 18 months post implant.

**Table 1:** Summaries for the 98 subjects included atbaseline

	Variable	Value n (%)
Sex	Female	43 (44.4)
	Male	55 (55.6)
Implant side	Left	32 (32.7)
	Right	66 (67.3)
Hearing loss type in implanted ear	Mixed	7 (7.1)
	Sensorineural	91 (92.9)
Hearing loss onset in implanted ear	Progressive	82 (83.7)
	Sudden	15 (15.3)
	Congenital(post-lingual)	1 (1)
Hearing loss severity in implanted ear (as per ASHA guidelines)	Moderate	2 (2.0)
	Severe	27 (27.6)
	Profound	69 (70.4)
Etiology	Unknown	60 (61.2)
	Otosclerosis	9 (9.2)
	Chronic Otitis Media	7 (7.1)
	Meniere’s	6 (6.1)
	Other	5 (5.1)
	Genetic	3 (3.1)
	Trauma	3 (3.1)
	Noise Exposure	2 (2.0)
	Ototoxic Drugs	2 (2.0)
	Meningitis	1 (1.0)
Pre-Implant Hearing aid (HA) use	Bilateral	70 (71.4)
	Left hand side	12 (12.2)
	Right hand side	10 (10.2)
	No HA	6 (6.2)
Highest level of education	Post secondary/tertiary	55 (56.1)
	Lower secondary education	23 (23.4)
	Primary education	17 (17.3)
	Pre-primary education	3 (3.1)
Current work status	Retired	76 (78)
	Working full time	10 (10)
	Working part time	6 (6)
	Voluntary not employed	3 (3.1)
	Other	3 (3.1)

ASHA - American Speech-Language-Hearing Association

Mean age (standard deviation) was 71.7 (7.6) (range 60–91) years, mean age at onset of severe hearing loss was 65.2 (12.3) (range 9–88) years.

### Outcomes

The data logging feature of the externally worn sound processor allows the clinician to see for how long the cochlear implant was activated during a day and in what sound conditions. This showed that devices were worn for a mean of 12 h per day (SD 2 h) with a minimum of 3 h mean daily use and maximum of 16 h. Most of the time when devices were used was spent in a quiet environment, followed by speech in noise.

Statistically significant improvements in scores were observed for all the measures at 18 months post implantation compared with baseline, except depression (Tables 2 and 3).

**Table 2:** Results of analysis of outcomes using linear mixed effects models (MEM).

Linear MEM outcomes	Baseline Mean (SEM)	Change from baseline to 12 month (95% CI)	Change from baseline to 18 months (95% CI)	Change from 12 to 18 months (95% CI)	Significance of overall effect of visit
HHIE-S	29.3 (1.08)	-8.02 (-10.44, -5.61)	-8.8 (-11.26, -6.34)	-0.77 (-3.25, 1.71)	F[2,159.04] = 45.12, p < 0.001
HUI3 Multi	0.40 (0.03)	0.14 (0.07, 0.20)	0.13 (0.06, 0.19)	-0.01 (-0.07, 0.06)	F[2,431.04] = 43.61, p < 0.001
HUI3 Hearing	0.31 (0.03)	0.23 (0.16, 0.31)	0.25 (0.18, 0.33)	0.02 (-0.05, 0.10)	F[2,439.56] = 106.83, p < 0.001
GDS-15	3.64 (0.36)	0.07 (-0.75, 0.89)	-0.16 (-0.99, 0.67)	-0.23 (-1.09, 0.63)	F[2,169.87] = 0.21, p = 0.81

Change from baseline to 12 and 18 months after implantation for measures using linear mixed effects models (MEM). All models adjust for age group and confidence intervals use a Tukey adjustment for multiple comparisons. Only the GDS-15 was not statistically significant. SEM: standard error of the mean. CI: confidence interval

**Table 3:** Results of analysis of outcomes using ordinal mixed effects models (MEM)

Ordinal MEM outcomes	Baseline Mean (SEM)	Odds of higher score: 12 months versus baseline (95% CI)	Odds of higher score: 18 months versus baseline (95% CI)	Odds of higher score: 18 months versus 12 months (95% CI)	Significance of overall effect of visit
HUI3 Hearing	0.32 (0.03)	9.50 (4.07, 22.20)	14.08 (5.65, 35.11)	1.48 (0.66, 3.32)	χ^2^ [2] = 68.86, p < 0.001
HUI3 Speech	0.87 (0.02)	3.14 (1.16, 8.54)	3.89 (1.36, 11.17)	1.24 (0.43, 3.55)	χ^2^ [2] = 12.07, p = 0.002
CAP II	4.98 (0.23)	14.81 (6.63, 33.15)	24.38 (10.28, 57.84)	1.64 (0.82, 3.29)	χ^2^ [2] = 105.50, p < 0.001
IADL	7.03 (0.12)	4.14 (1.70, 10.12)	3.40 (1.40, 8.24)	0.82 (0.32, 2.09)	χ^2^ [2] = 18.31, p < 0.001
DJLS	2.19 (0.17)	0.55 (0.28, 1.08)	0.38 (0.19, 0.78)	0.70 (0.34, 1.42)	χ^2^ [2] = 10.75, p = 0.005

Odds of a higher score between time points for measures using ordinal MEM models. All models adjust for age group and confidence intervals use a Tukey adjustment for multiple comparisons. SEM: standard error of the mean. CI: confidence interval

The gains exceeded the clinical significance thresholds for the HUI3 multi-attribute index, and for the single attribute domains for speech (ability to be understood) and hearing (ability to hear).

Baseline mean speech attribute scores were indicative of moderate disability and changed to mild or no disability post implant. Hearing domain and multi-attribute scores were still indicative of severe disability post implant. Sixty-one (62%) subjects gained at least 0.03 on the HUI3 multi-attribute index, representing a clinically noticeable improvement in health utility after implant.

**Fig. 1:**
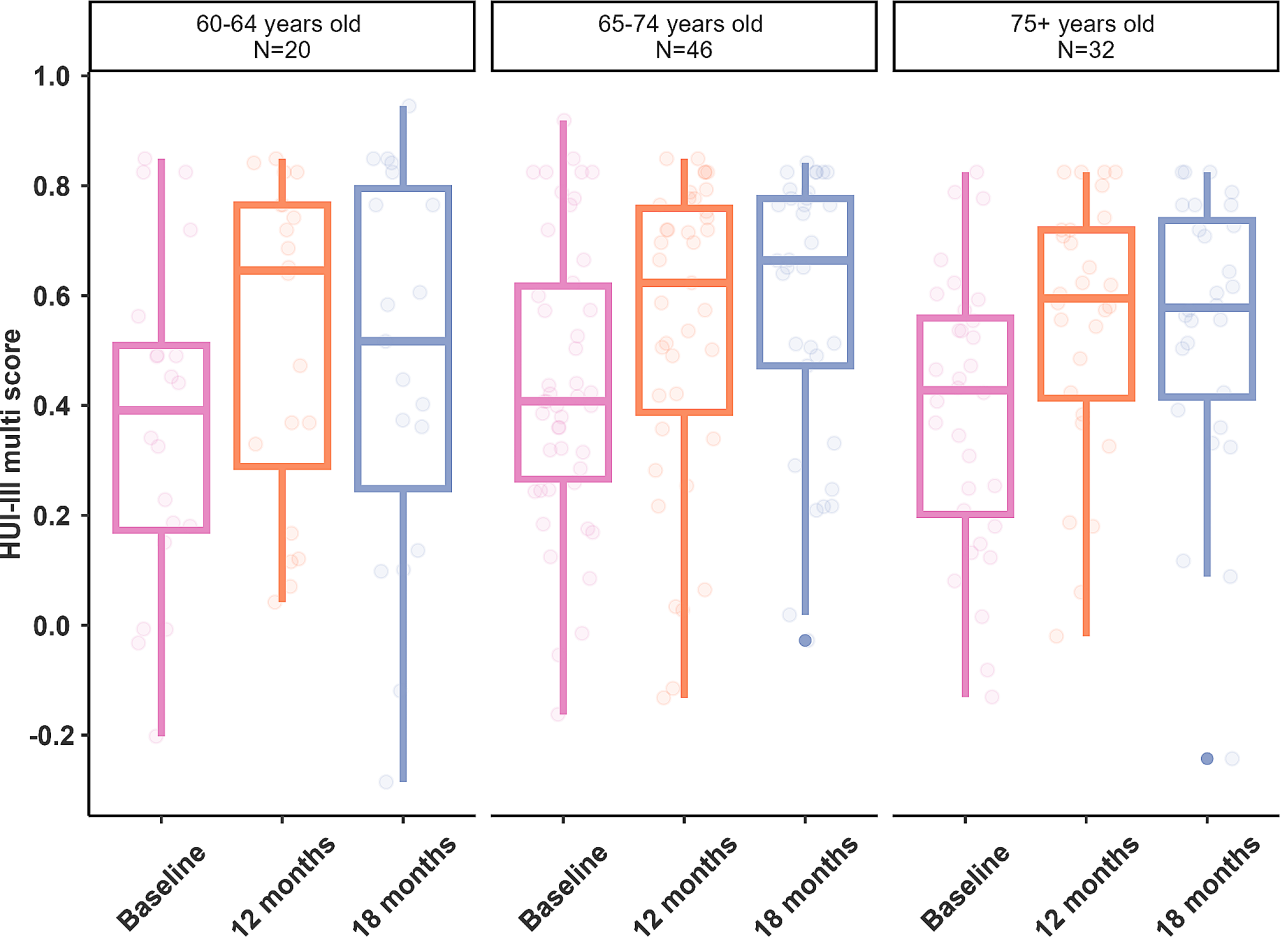
Box plot showing scores for the HUI3 for each age category. There was no significant difference in scores by age group. Boxes show 1st quartile, median and 3rd quartile; error bars 1.5 time the interquartile range; and dots outliers. Normal values for 60–74 years olds are 0.8 and for 75 + year olds are 0.7

There was no statistically significant difference in the HUI3 multi-attribute index between the 60–64, 65–74 and 75 + years age groups (F[2,9228] = 0.53, p = 0.59) (Fig. 1). Age was also not a statistically significant factor for any of the other measures (p > 0.20).

**Fig. 2:**
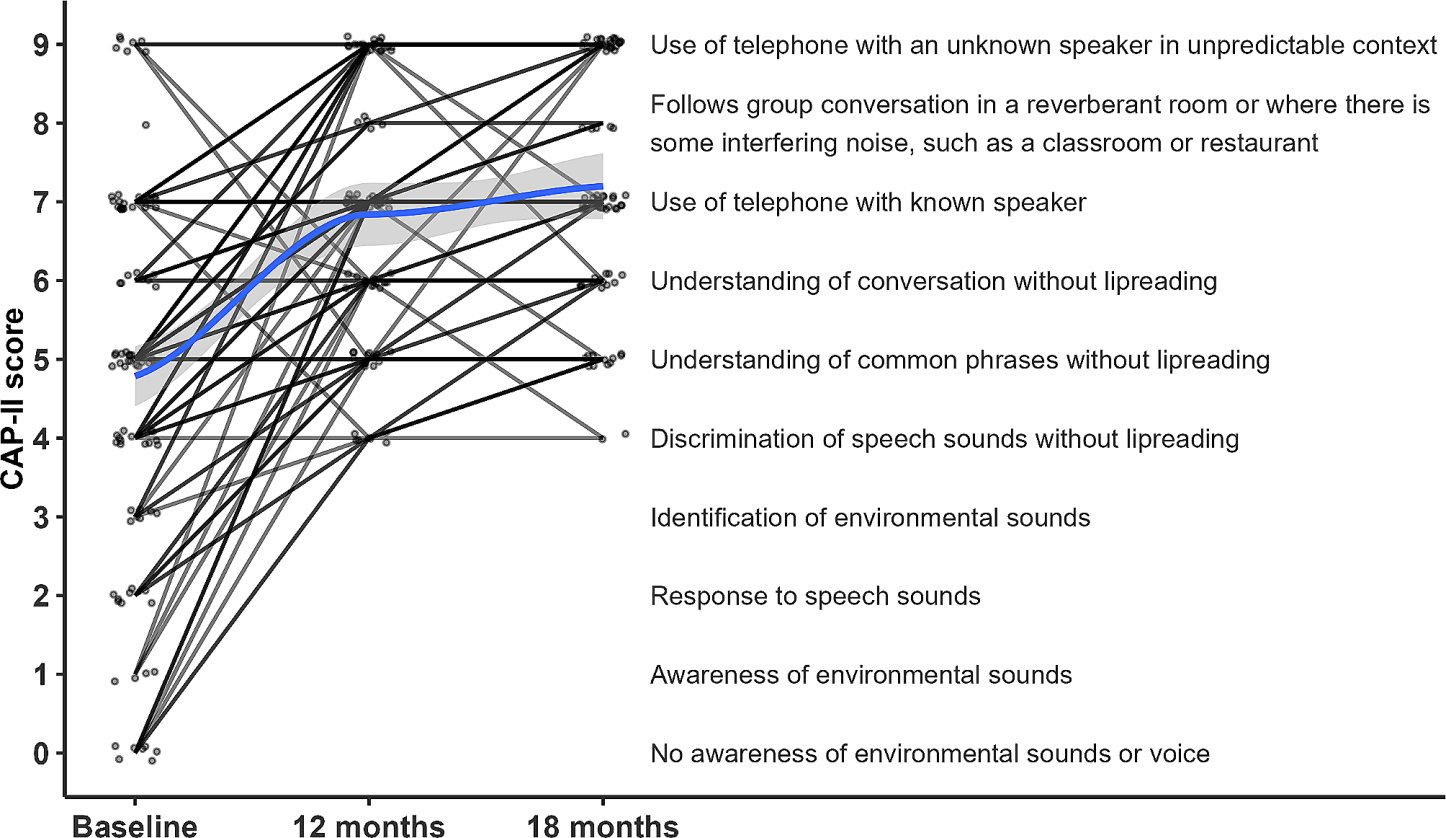
CAP-II scores for individual subjects. Solid line represents the median value and shaded area the 95% confidence interval

The disease specific measures of hearing function, the CAP II and HHIE-S both showed large statistically significant gains. Median CAP II scores at baseline corresponded to “understanding of common phrases without lipreading”. This improved by 18 months to indicate “use of telephone with known speaker” (Fig. 2). All subjects achieved a level of speech sound discrimination without lip reading post implantation.

**Fig. 3:**
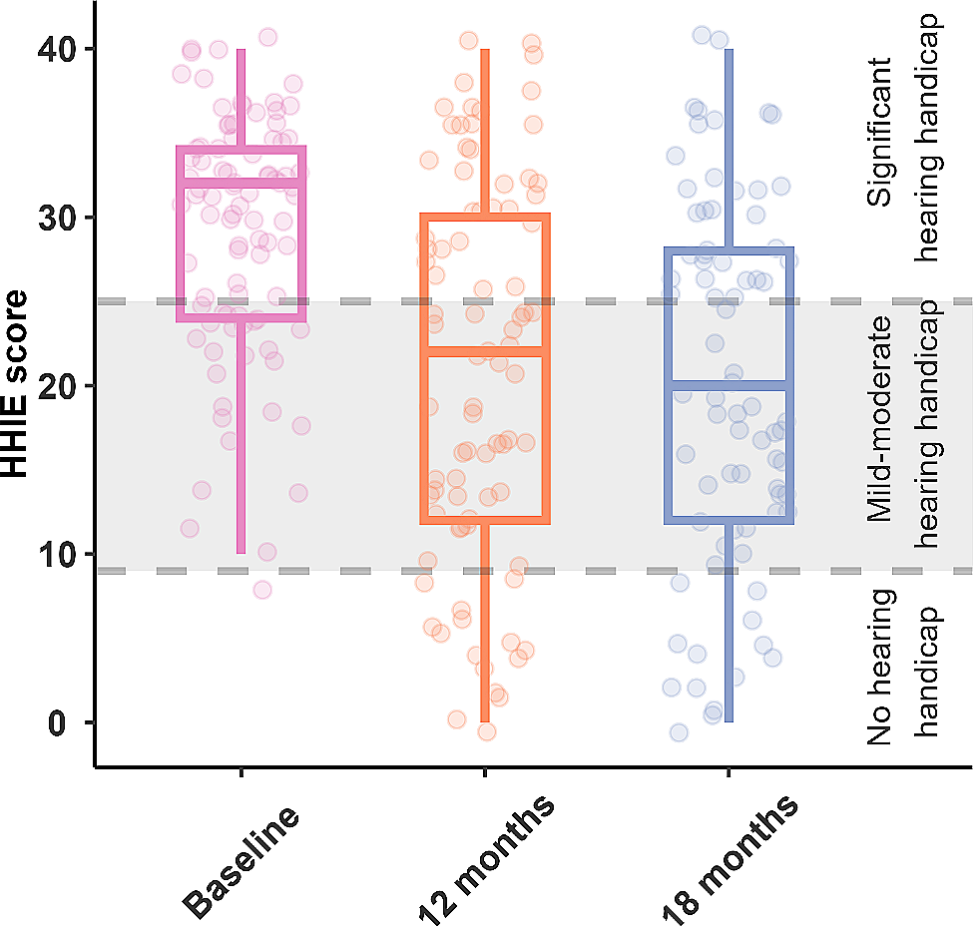
HHIE-S scores. Lines/shading indicates handicap boundaries. Scores of 0–8 suggest no hearing handicap 10–24 suggest mild-moderate hearing handicap 26–40 suggest significant hearing handicap. Boxes indicate 1st quartile, median and 3rd quartile; error bars 1.5 times the interquartile range

**Fig. 4:**
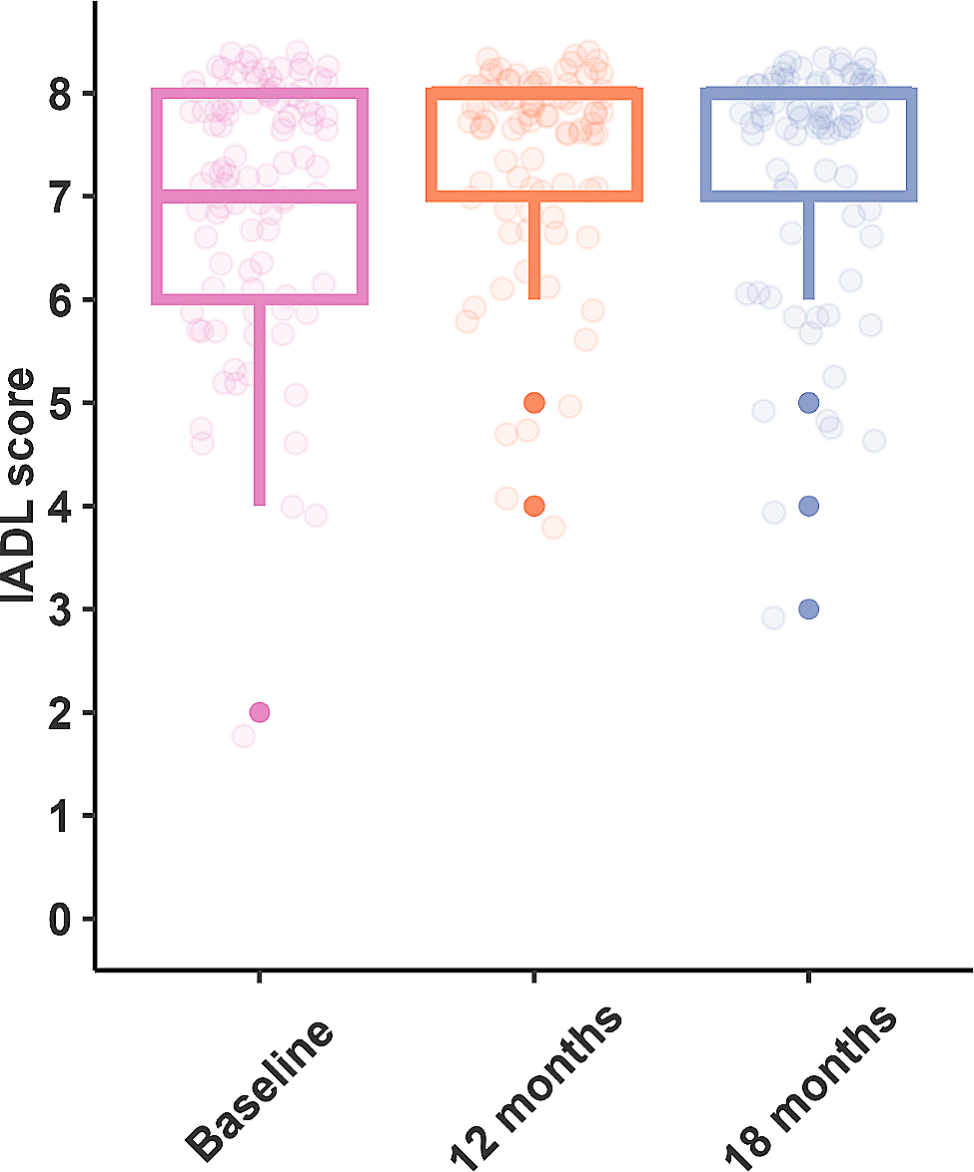
Box plot showing scores for the IADL. By 12 months post implant most individuals were achieving the highest score on this test. Boxes indicate 1st quartile, median and 3rd quartile; 1.5 x interquartile range and bold dots outliers

**Fig. 5:**
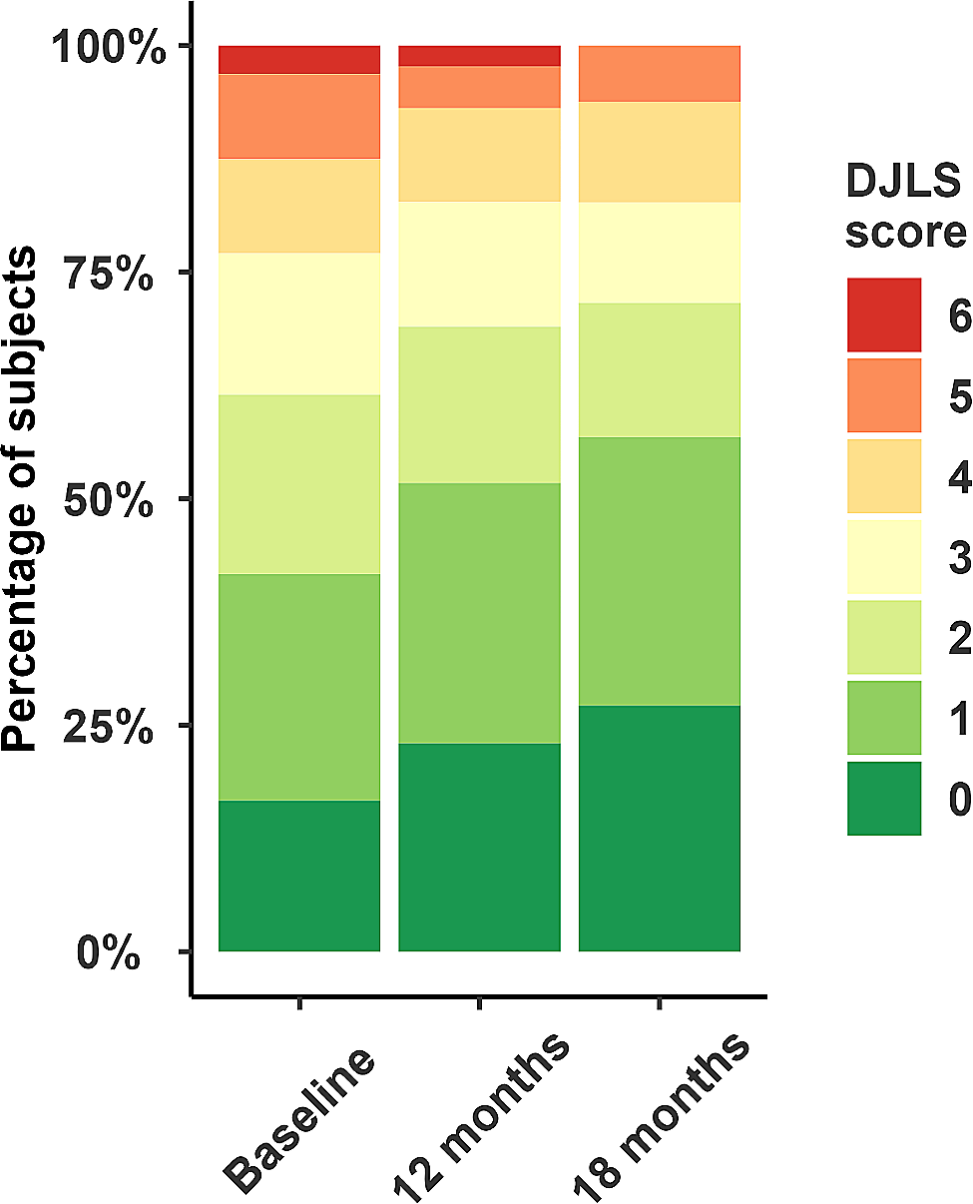
De Jong Loneliness scores by visit. The plot shows proportions of subjects with each score. Scores of 0 and 1 are considered as “not lonely”; those > 1 as “lonely”. Between baseline and 18 months subjects became significantly less lonely (p < 0.01)

Mean and median group HHIE-S scores moved from being classified as a significant hearing handicap before implant to mild-moderate hearing handicap after 18 months of implant use (Fig. 3). However, the mean gain of 8.8 points was just outside the clinically significant range (9 points). The hearing handicap category changed after implant for 37 subjects, thirty-five (36%) individuals moved to a lower category after implantation and two (2%) individuals moved to a higher hearing handicap category. Scores for daily living indicated a significant improvement in independence at 18 months. A ceiling effect was reached by at least 50% of subjects by 12 months after implant (Fig. 4). Odds of higher loneliness scores was significantly lower at 18 months post implantation compared to baseline (OR 0.38, 95%CI 0.19–0.78). The proportion of subjects reporting scores of > 1 indicating loneliness, reduces over time (Fig. 5).

The mean score for the GDS-15 at baseline indicated no depression and this did not change after implantation. The percentage of individuals with depression did not change over time either (Table 4).

**Table 4:** Percentages of individuals with depression based on scores on the GDS-15 at each interval

	No depression	Mild depression	Moderate depression	Severe depression
Visit 1	72%	18%	7%	3%
Visit 2	69%	17%	8%	6%
Visit 3	70%	24%	2%	4%

Scores of 0–4 are considered normal, scores of 5–8 indicate mild depression; 9–11 indicate moderate depression; and 12–15 indicate severe depression

## Discussion

There is currently a lack of robust data on health utility changes following cochlear implantation in older adults. This study is the first prospective international study in elderly CI users with health utility as its primary outcome measure. It provides important cross-cultural data from a large sample for this age group. The large statistically and clinically significant multi-attribute health utility gain of 0.13 reported here is within the range for unilateral cochlear implantation for older and younger age groups [17]. In a recent meta-analysis, a pooled mean HUI3 gain of 0.17 (95% CI 0.12 to 0.23) was reported for unilaterally implanted adult subjects where mean ages ranged from 37 to 77 years [17]. There was no significant difference in HUI3 gain across our young-old to old-old age groups, in line with other studies where HUI3 gain was not significantly different between age groups ranging from 18 to 34 years to 65 + years [24]. The gains observed after 18 months of CI use were four times the minimum gain suggested for a noticeable difference to the individual. Results supersede the three underpowered smaller studies, indicating that older adults do not get health utility gains [14, 18, 20]. The small size of the samples in these earlier studies likely influenced the ability to demonstrate significance and the clinical relevance of the results observed. The gains observed in the HUI3 in this study may have been limited by the inherent ceiling effect that exists in the HUI3 for the hearing scale [15]. Most CI recipients are simply unable to progress beyond rating level 3 (able to hear what is with a hearing aid) as they still rely on their implant device to hear. This aspect of the HUI3 currently limits its sensitivity to changes in hearing function. Work is being done to improve the sensitivity of the hearing domain within the HUI3, that ultimately may better estimate the utility gains from hearing treatment [17].

At 12 months post implant all participants could at least discriminate speech sounds without lipreading using their CI, despite some having no awareness of environmental sounds or voice at baseline using hearing aids. By 18 months at least 50% of subjects were able to use the telephone and 25% were able to score at normal-hearing levels for this test. This improvement in hearing was reflected in the clinically and statistically significant reduction from significant to mild-moderate hearing handicap after implantation. Subjects also reported an improvement in their ability to be understood, with a clinically and statistically significant change in the HUI3 speech attribute scores. Mean scores 18 months after implantation in indicated no remaining disability in this area. The amount of time the sound processor was used by each individual was recorded by the data logging feature. This showed that device use was high, with participants using their devices for an average of 12 h per day. There were no subjects who chose not to use their implant.

Scores on the GDS-15 scale indicated that the majority of participants were not depressed. The average percentage of those with mild to severe depression was in line with that expected for an elderly population and did not change during the course of the study [37].

Other related quality of life measures also showed statistically significant improvements for the group after implant. A significant reduction in loneliness scores was observed by 18 months. At baseline, mean values were slightly poorer than normal values reported for European countries, as expected for hearing impaired individuals [5, 30]. After implant, mean scores were brought within the normal range. These results are in line with literature reporting reduced reports of loneliness after implant compared with before implant with a hearing aid [38]. The IADL scale showed that participants were more independent after implantation: At 12 months post implant, more than 50% of subjects were at the highest level of independence on this measure. Greater levels of independence following implantation are also reported in the literature: Sonnet and colleagues (2017) reported an improvement in a small sample of 16 subjects using the same IADL scale and Völter and colleagues (2018) showed a benefit in the autonomy-related questions of the WHOQOL-OLD questionnaire [10, 39]. However, Sarant and colleagues (2019) reported no change with the Bayer activities of daily living scale. Inspection of the individual areas showed that this change mostly came from the 15 individuals scoring 0 at baseline on the telephone scale (Does not use telephone at all) and scoring 1 post implantation (some telephone use at varying levels) [18]. There was little change for other questions.

The importance of telephone use and its impact on quality of life is highlighted in Müller and colleagues (2022), where those who were unable to use the telephone before implant were 1.5 times more likely to get a meaningful gain in health utility after implant [15]. Better hearing, including the restoration of the ability to use the telephone may have contributed to the observed reduction in loneliness. This may in turn have important implications for delaying cognitive decline [11].

Age was not a factor in this older group of subjects for any of the measures suggesting that younger and older groups do not differ in the benefit gained, supporting the assertion that age alone should not be a barrier to receiving an implant.

Further research should focus on how better hearing ability, reduced social isolation and loneliness and greater independence may impact cognitive decline and dementia. Particular attention should be given to the roles of maintaining social connections and independence via telephone use and other digital modes of communication. Improved health utility measures are required to better estimate the impact of changes in hearing function on overall quality of life.

### Limitations

Each participant was required to answer many questionnaires during each evaluation visit, which may have led to fatigue and subsequently less accurate responses. Some of the study data was collected during a period where Covid-19 pandemic social distancing restrictions were in place. This may have limited both the number and type of situations experienced and influenced the responses given. It may also have led to a decrease in the overall quality of life and an increase in reports of loneliness. The inclusion of more sensitive disease specific quality of life measures may have shown greater gains in quality of life [24]. There was no prolonged follow up beyond 18 months, hence the effect of more time upon observed benefits is unknown. Many of the measures used were developed as screening tests, and have variable sensitivity and thus may not be diagnostic of a particular condition e.g. depression. Only the HUI3 was validated in all the languages used. Although only subjects using cochlear implant devices manufactured by one company were recruited, it is expected that results would be applicable to all CI users regardless of device manufacturer.

## Conclusions

After receiving a cochlear implant, the quality of life of this group of adults ≥ 60 years old improved significantly. Participants wore their devices for an average of 12 h per day. Better hearing not only improved individuals’ ability to communicate verbally, but also their ability to function independently on a daily basis. They appeared to feel less lonely and felt less handicapped by their hearing loss. Clinically and statistically significant gains in health utility, independence, loneliness, hearing ability and hearing handicap were observed regardless of young, middle or old old age group. Cochlear implants contribute to overall wellbeing for healthy ageing and should be considered as a routine treatment option for those aged 60 years or over with bilateral moderately severe to profound hearing loss.

## Data Availability

Deidentified individual participant data and relevant study documentation used in this study are available upon reasonable written request to author CJJ (cjames@cochlear.com).
